# Context Matters: Extra-Personal Factors Underlying Concussion Reporting in University Athletes

**DOI:** 10.3390/sports13030077

**Published:** 2025-03-05

**Authors:** William Archambault, Dave Ellemberg

**Affiliations:** 1 École de Kinésiologie et Sciences de l’Activité Physique, Faculty of Medicine, Université de Montréal, Montreal, QC H3T 1J4, Canada; dave.ellemberg@umontreal.ca; 2 Ingram School of Nursing, Faculty of Medicine and Health Sciences, McGill University, Montreal, QC H3A 0G4, Canada

**Keywords:** concussion disclosure, qualitative methods, high-stakes vs. low-stakes contexts, sport, psychology

## Abstract

Gaps remain in our understanding of *which* factors contribute to concussion disclosure and *how* they contribute to this process, thereby limiting our ability to improve disclosure. This study aimed to characterize the most relevant extra-personal determinants of SC disclosure and to describe their influence on the disclosure process. To that aim, the first author conducted substantive qualitative interviews with nine university student–athletes and analyzed their content via constant comparative analysis (guided by Straussian grounded theory). Eleven (11) extra-personal concepts influencing concussion reporting were identified and described across two categories: *Contextual Incentives* and *Socio-Cultural Pressures*. These findings suggest that each identified concept can individually shape the context around the injury, creating either higher-stakes conditions that deter disclosure or lower-stakes conditions that encourage it. Further, the results posit that these concepts interact and collectively influence athletes’ decision-making process by modulating the perceived stakes of disclosing a concussion. If these findings hold true in more diverse populations and contexts, they suggest that adapting concussion prevention efforts to consider these contextual variables could improve SC disclosure. This study also highlights the benefits of using qualitative methods in the investigation of concussion reporting.

## 1. Introduction

Over recent decades, the breadth of knowledge generated from scientific inquiry on sport concussion (SC) has improved our understanding of this injury and led to many positive outcomes [1]. For example, although the incidence of SC is rising, it appears to be a result of enhanced awareness and improvements in diagnostic tools and skills [2,3]. Today, when a SC is detected, most athletes undergo a specific management protocol, and the injury usually resolves within 2–4 weeks without significant complications [4,5,6,7].

Science has also taught us much about the negative health consequences of SC. They can produce debilitating and even life-threatening sequelae when they are mismanaged or if they remain undetected [5,8]. From a public health perspective, the best strategy to reduce the incidence and burden of SC is to remove athletes from sport activities as soon as a concussion is suspected. This allows for the assessment of the injury and, if a concussion is confirmed, ensures athletes follow the appropriate recovery guidelines [8].

Despite advances in clinical imaging and in cognitive testing, the diagnosis of SC remains heavily dependent on symptom disclosure [8]. This is disquieting given a significant proportion of sports concussions still go undetected due in large part to non-disclosure of symptoms by a significant proportion of athletes from various backgrounds [9,10,11,12].

To facilitate concussion symptom reporting, it is necessary to identify and characterize the factors influencing disclosure and explain their role within this decision-making process. Many studies have begun to catalogue some of these factors, but they are not without limitations [10,11,13,14,15,16]. For one, athlete-generated insights remain inadequately considered because most studies use surveys that are made up of pre-selected and closed-ended questions, which are limited in their ability to fully identify and describe the processes underlying an athlete’s decision to disclose or not their SC. Furthermore, the phenomenon of *satisficing* is known to affect the quality of survey responses [17]. That is, respondents will invest the minimal satisfying effort when completing a task rather than reflecting and thinking through every possible answer. In studies of concussion underreporting, *satisficing*—a decision-making shortcut where individuals opt for an acceptable but not optimal answer—can be observed when participants rarely choose the “other” option from a list of justifications for non-disclosure, or when they select only one justification, even though multiple reasons may apply [10,18]. This behavior suggests that participants may prefer the cognitive ease of choosing a single, straightforward response, rather than thoroughly considering or articulating more nuanced explanations for their decision not to report concussions.

One way to overcome these limitations is to use qualitative methods of data collection and analysis as they are less prone to *satisficing* and can produce rich, participant-generated descriptions of events [19,20,21,22]. Qualitative investigations revealed novel determinants of SC disclosure not identified in survey studies and they provide enriched details regarding the influence of known determinants of SC disclosure [23,24,25,26].

Despite their aforementioned benefits, qualitative studies are relatively scarce in this area and come with limitations. For instance, interviews are frequently overly structured, relying on questions derived from surveys employed in prior research. Moreover, these studies tend to focus on established factors driving disclosure, thereby skewing the data collection towards intra-personal determinants. Consequently, there is a paucity of research on extra-personal determinants of SC disclosure [23,25,26,27]. Some such extra-personal and contextual determinants of SC reporting have been identified in previous studies. These include: ‘*trust in medical personnel*’ and ‘*pressures from coaches, teammates, and close relatives to pursue competition*’. However, studies fail to systematically distinguish between intra- and extra-personal determinants of SC reporting and fail to incorporate them into a holistic and coherent model of SC disclosure [16,23,25,28,29,30]. This warrants a more thorough examination of extra-personal determinants of SC disclosure to uncover which ones are most impactful as well as their possible mechanistic influence on the disclosure process.

For example, previous work has suggested that concussion-naïve athletes have a tendency toward disclosing their first concussion but then quickly adopt a ‘*non-disclosure bias*’ for subsequent ones. The disclosure of a future SC occurs only if it is judged to be worse than their prior worst SC. This personal ‘disclosure threshold’ appears to be modulated by intra-personal factors such as ‘status on the team’, ‘maturity level’, ‘prioritizing athletic over intellectual activities’, and ‘cultural knowledge of concussions’ [22]. However, this study is limited by its exclusive focus on intra-personal determinants of SC disclosure. Gaps remain regarding which extra-personal factors contribute to SC disclosure and whether they also operate through the adoption of a ‘*non-disclosure bias*’ or via other mechanisms. Exploring these gaps is important as they could guide future research on SC reporting and the elaboration of assessment tools aiming to promote disclosure and detection of concussions.

Using qualitative interviews and grounded theory, the objective of the present study is to identify the extra-personal factors influencing sport concussion disclosure and characterize how they influence the decision-making process underlying reporting behaviors.

## 2. Materials and Methods

### 2.1. Design

The following sections expands our methods described in previous work with the same population [22]. Given the objectives of this research, and because of its emphasis on processes and contextual variables, Straussian Grounded Theory (GT) was identified as the most appropriate qualitative approach to identify and analyze variables influencing concussion disclosure in athletes [20].

### 2.2. Ethics and Demographics

Our exploratory qualitative approach used a mixture of criterion and purposive sampling. Following approval of the research protocol by the Institutional Review Board (IRB) of the authors’ university, we applied criterion sampling by recruiting student-athletes engaged in sports associated with concussions. This ensured that we interviewed individuals possessing knowledge and experience relevant to our research focus. Participants without a history of concussion were intentionally encouraged to participate in our study as their perspective is important to the understanding of concussion reporting and is absent from the current literature on the topic [23]. Finally, as per Straussian GT, theoretical sampling was maximized to achieve saturation of the concepts and categories emerging from the analysis.

A total of nine university student-athletes representing three sports (five females and four males; Soccer, Rugby, and Cheerleading) answered our call and consented to being interviewed (see Table 1). At the time of the interview, none of the athletes with a history of concussion (HoC) was following a concussion recovery protocol. To preserve confidentiality, pseudonyms are used when presenting quotes from the participants.

### 2.3. Data Collection and Procedure

Following IRB approval, calls for participants were sent to the university sport teams. Interested participants reached out to the research team and, after obtaining informed consent, a meeting was scheduled for the interview. Prior to the first interview, the authors designed an open-ended interview guide to conduct in-depth, semi-structured interviews. The initial interview guide was designed with both the aims of the research project and summary knowledge of the current literature on concussion reporting. Although proponents of the classical (Glaserian) GT argue that prior literature knowledge should be avoided to limit biases in the data collection and analysis, Straussian GT views summary knowledge of the scientific literature as a means to develop theoretical sensitivity. This facilitates identification of relevant concepts and their integration into a more appropriate model [31].

The first part of the interview gathered information on participants’ history of concussion and general knowledge of concussion. Next, participants were asked to recall their past concussion(s) and discuss the reporting process (e.g., *Did they disclose their symptoms, yes or no? Why or why not? How and to whom? What was the context? Etc.*). For participants who reported no prior concussion, questions were asked in a hypothetical form (e.g., *What would you do if you were to get a concussion next season?*). Finally, specific questions related to the components of the TPB (attitudes, perceived subjective norms, and self-efficacy) and the influence of one’s entourage (e.g., therapist, coaches, teammates, family, etc.) were included given they had been previously identified as moderate influencers of concussion-reporting behaviors in athletes [29,30].

In line with Straussian GT procedures, analysis began after the first interview and proceeded in an iterative fashion, following principles of theoretical sampling [20]. Concretely, it means that data collection and analysis occurred in parallel, and the interview guide was refined after each interview to accommodate purposeful exploration of novel concepts or gather more data on concepts and categories requiring further saturation. To respect the participant’s availabilities, analysis of the previous interview was not always possible before the subsequent one. In such cases, the interviewer listened to the audio files shortly before the next interview, paying close attention to any relevant new insights and concepts to be incorporated within the interview guide. Examples of topics that were added to the interview guide following the first few discussions include (i) the influence of school authorities and social media; (ii) professional sport ambitions; and (iii) the role of culture and religion.

Data collection took place between May and August 2020, amidst the COVID-19 pandemic. Nine semi-structured interviews (range = 56–79 min; total = 587 min) were conducted remotely and recorded using the Zoom platform [32]. Although it may seem like a small number of participants, this is quite common in qualitative research [23,33]. Instead, constant comparison analysis inspired by grounded theory is more interested in data quality than quantity. Achieving conceptual saturation, meaning sufficient data collected to properly define and describe concepts and categories, is considered a good measure of data collection quality [20,34]. Hence, recruitment stopped after the ninth interview given that no new concepts had emerged from the last two interviews and that most concepts were deemed to be sufficiently saturated. To maximize conceptual saturation after the final interview, emails were sent to all previous participants to provide them with an opportunity to comment on concepts and categories that emerged after their interview.

All interviews were conducted, transcribed, and analyzed by the first author. Save for one (English), all interviews were conducted in French. Citations were translated to English by the first author and reviewed by the second author.

### 2.4. Data Analysis

Verbatims of the interviews were uploaded, coded, and analyzed using QDAMiner 6 Software [35]. The analysis adhered to the procedures outlined by Strauss and Corbin, which are briefly summarized below [20].

The first step of analysis, known as ‘open coding’ or ‘line-by-line analysis’, involved analyzing interview transcripts, sentence by sentence, to extract concepts. Concepts can be a word or short phrase representing a first-level abstraction of the meaning of the qualitative data. Extracted concepts are what guide the adjustments to the interview guide in the process of theoretical sampling referred above. Each new concept is constantly compared with those already identified to refine their definition and meaning. In the context of this study, concepts represent contributors to SC disclosure.

When sufficient data were gathered to produce a significant number of well-defined concepts, the second step of the analysis consisted in grouping them into a second level of abstraction called categories. Categories can then be described in terms of their properties (i.e., the concepts composing them) and dimensions (i.e., variations in their properties).

The final step of the analysis, known as ‘axial coding’, occurred once several categories emerged from the data and consisted in reanalyzing the original raw data to establish links between the categories and the concepts supporting them. In line with Straussian GT, these links were established by exploring data for context, initial conditions, action and interaction strategies and their consequences [20]. These links were supported by data and by observations and reflections of the researcher, described and archived into written memos [36].

In addition, extra steps were taken by the authors to improve the quality and validity of the analytic process. Random samples (i.e., 10%) of all codes were counter-coded by a non-author collaborator, and a concordance level of 87% was achieved. For the few cases where there was disagreement, short discussions allowed to modify certain code attributions or to refine the definition of certain concepts. Additionally, the coauthor and non-author colleagues acted as critics and provided feedback into the definition, description, and relationship of the concepts and categories.

## 3. Results

We identified eleven (11) concepts divided into two key extra-personal categories: *Contextual Incentives and Socio-Cultural Pressures*. Descriptive summaries of these concepts are presented in Table 2 and will be further detailed in the following sections.

### 3.1. Contextual Incentives

All participants agreed that some settings were more favorable to disclosure than others. Significant contextual factors identified within our data are the subjective importance of the moment, the athlete’s perceived influence on the outcome of the current competition, the timing of the decision, whether the injury occurs during an athlete’s final season, and some sport-specific realities.

#### 3.1.1. Subjective Importance of the Moment

Unanimous among our participants is the fact that some competitions or contexts are more valued than others. For example, all athletes attributed more worth to a championship game or a final competition than a practice or a regular season game. In other circumstances, the importance of an event is more personal, such as when a player knows there is a recruiter in attendance. Disclosure likelihood drops as the importance of the moment increases for a given athlete:

***Bianca:*** *“When I disclosed my symptoms, it was because it happened during a practice. For me, games are more important and there were not that many left to the season. Whereas practices, there are many more and you can make up for them.”*

***Evelyn:*** *“This year we reached the semi-final for the very first time. It was a great team achievement and lots of fun. If I had gotten a concussion, I don’t think I would have disclosed it. I would not have wanted to miss this experience!”*

#### 3.1.2. Perceived Influence on the Outcome of the Competition

Our data also indicate that an athlete’s perceived influence on the outcome of a competition weights significantly in their decision to disclose their injuries. Indeed, athletes would be more inclined to report concussion symptoms if the injury occurs when the outcome is already determined. One participant said it could even negate the effect of the importance of the game:

***Georges:*** *“If a team is losing by several goals with just a few minutes left, even if it is the championship, I think most would say something and get out of the game. It’s not worth the risk when you won’t have an impact on the game.”*

#### 3.1.3. Timing of Decision

Our data also suggest that the timing of the concussion can influence the decision to disclose. Athletes mentioned that disclosure would be more likely after or in-between competitions, when they have time to think and assess their situation. Conversely, game-time decisions are more instinctive, emotional, and centered on the present, favoring non-disclosure:

***Anthony:*** *“When I speak to you right now, it is obvious that the right decision should be to disclose to avoid making things worse. But in the heat of the moment, you don’t think about that. Your reflex is to think about getting back on the field.”*

#### 3.1.4. Final Season/Year

Veterans playing their last university season would also be at higher risk of under-reporting concussion symptoms compared to veterans with one season or more left. Several of our interviewees mentioned that the final season is a final opportunity to appreciate their university career, win a championship, or impress professional scouts. However, other participants nuanced this claim by reporting that certain graduating athletes could be more careful and conscious of their health during this final season to avoid jeopardizing their post-university career.

***Inna:*** *“For 5th year veterans (final year of University Sport in Canada), it may be their last chance to compete in their sport at a high-level. There is also probably some pride and wanting to prove yourselves or exhibit toughness. You don’t want to spend your last year on the sidelines.”*

***Anthony:*** *“Towards the end of their university careers, some veterans start to project themselves beyond the field and into the next stage of their life. They become more conscious of their health risks, and it might make them more prone to disclose their symptoms.”*

#### 3.1.5. Sport-Specific Realities

Lastly, it appeared that certain sport-specific features can weight significantly on the decision to report or not concussive injuries. This contextual property was most salient for our cheerleading participants, notably in the design and rules of their competitions. Unlike most team sports, cheerleading teams do not face a single adversary on the same turf. Competitions involve multiple teams taking turns performing an acrobatic routine lasting approximately two or three minutes in front of judges. As pointed out by three cheerleading athletes, the short duration of the performance, the time invested in learning a precise routine, and the difficulty in finding replacements in cases of injury are very strong incentives against disclosing concussion symptoms:

***Hannah:*** *“The thing is that if you’re injured, the rest of the team can’t practice the routine because everyone has a specific part to play, and it doesn’t work unless everyone is there. It is very complicated to find a substitute who can do the exact same thing as you. So, coaches don’t have incentives to take someone out. Your teammates beg you to push through. And you tell yourself: ‘It’s just 2–3 min. I’ll tough it out and I’ll deal with it after.’ At competitions, it is quite common to see people struggle to get off the mat, bleeding or missing a tooth. You don’t want to be the one that makes everyone else stop’.”*

#### 3.1.6. Summary of Contextual Incentives

**Figure 1:**
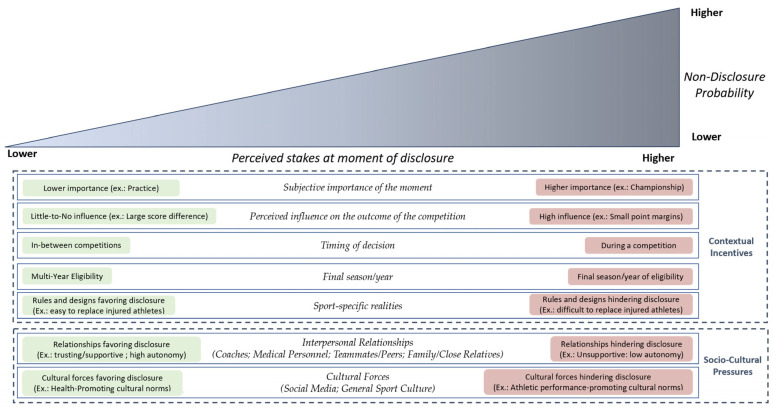
How perceived stakes influence non-disclosure of concussions symptoms.

In brief, our athletes suggest that concussions are less likely to be reported if they happen in the middle of an important competition with undetermined outcomes, during an athlete’s final season, and in sports involving technical and short routine performance. In comparison, practices, regular season games without much significance on the rankings, contests with large point differentials, or symptoms appearing in between competitions are all situations during which concussion disclosure would be more probable. All these varying influences are depicted within Figure 1.

### 3.2. Socio-Cultural Pressures

All participants reported that socio-cultural pressures can either facilitate or hinder concussion reporting. Sources of socio-cultural influence can be relational, such as an athlete’s relationship with their coaches, medical personnel, teammates/peers, and family/relatives, or stem from cultural norms such as those conveyed by social media and the general sport culture.

#### 3.2.1. Coaches

Due to their decisional power in terms of roster and playing time, coaches were viewed as having a significant influence on concussion disclosure:

***Evelyn:*** *“This social pressure can come from coaches, and it can go either way. I had a coach who was really focused on health. At the smallest symptom or sign of injury you were out. While my other coach, I wouldn’t say he would make us play with a concussion, but he was very competitive and would maybe encourage us to play through certain injuries more than the average, which may make some athletes hesitate longer before coming to them with concussion symptoms.”*

#### 3.2.2. Medical Personnel

Also, no matter the role of the other person, our participants stressed the importance of trust in favoring honest communication. Concerning medical personnel, most participants stated that having a specific team therapist (rather than having therapists rotate among several teams) could promote disclosure by facilitating the development of a trusting relationship. However, some athletes expressed hesitance to disclose concussion symptoms because of the automatic withdrawal from play integral to current concussion management protocols. In response, some athletes admitted consulting with third party medical professionals, unaffiliated to the university or team, to maintain some control over that decision. For them, non-disclosure was a means to preserve autonomy and self-manage their injury:

***Darya:*** *“I know some teammates who got injured and they went to see an external third-party physician to avoid our team therapists knowing about it. It’s true that our therapists are quick to tell our coaches we cannot practice, even for the slightest ache.”*

Contrary to some beliefs, most participants felt that the influence of teammates is generally towards promoting disclosure and taking care of their health. Athletes do share details and concerns about their injury with each other and many of our participants reported either encouraging a teammate or having been encouraged by a teammate to disclose injuries, including concussions. However, they acknowledged that it is easy to recommend others to ‘do the right thing’ when they themselves have nothing to lose and that they probably would not have disclosed the injury if the roles were reversed.

***Hannah:*** *“In general, between us teammates, we are there to support and check on each other. But it is easier to encourage someone else to be careful and to report their injury than to follow this same advice ourselves when we hear it.”*

#### 3.2.3. Teammates/Peers

Although most participants agreed that, in theory, parents/close relatives have an influence on concussion reporting behavior, only one of our interviewees experienced it directly. Her experience supports the impression shared by other participants that parental influence tends to manifest as concerns for their children’s health. However, these worries also tend to backfire and favor non-disclosure:

***Inna:*** *“After each concussion I had, my mother would tell me to quit cheerleading because it is too dangerous. So, if I had concussion symptoms in the future, I probably would not tell her unless they were really bad. I don’t want to worry her or cause unnecessary friction.”*

#### 3.2.4. Social Media

The remaining two types of pressures within this category, social media and sport culture, are distinct from the others because they are cultural, rather than interpersonal. The impact of social media was marginal in our data since only one participant reported it could play a role. When deliberately questioned on its potential impact on concussion disclosure, other participants responded negatively or were uncertain. However, it did affect at least one person, and its influence may be different based on age, sport, institution, or level of notoriety of the individual.

***Evelyn:*** *“At my school, they sometimes post profiles of players on the school’s website. Last year, they did one on me stating that I was the first rugby player from the school to go on to play in university. That can put undue pressure on the athlete. After that, if I had had a concussion that would have prevented me from playing, I know I would have felt terrible to let down my school and would have been less comfortable disclosing it.”*

#### 3.2.5. General Sport Culture

In terms of sport culture, most participants acknowledged that they grew up in sport environments that encouraged and rewarded ‘playing through injuries’ or ‘acting tough’ and that this influences athletes to varying degrees. In addition, some mentioned that this ‘warrior mindset’ is not equally present for every sport or even across teams for a given sport:

***Inna:*** *“There is a difference between having a concussion in a university versus a civilian cheerleading team. […] In a civilian team, if you can still perform, you must. They won’t question you too much. In a university team, you’re automatically taken out. They won’t take a chance. So, girls who used to cheer in a civilian team before coming to university don’t like that and they won’t disclose their symptoms unless they really can’t perform.”*

#### 3.2.6. Summary of Socio-Cultural Pressures

As illustrated in Figure 1, the aggregate of all these socio-cultural interactions can create conditions that encourage or hinder concussion disclosure. Our data suggest that having coaches who promote health above performance, trusting and supportive relationships with therapists, teammates, and parents, and growing up or competing in a milieu that rejects the sport culture of toughness usually facilitates disclosure. Conversely, athletes would be less likely to disclose their concussion symptoms in socio-cultural environments where they feel a lower sense of autonomy and control over their participation in physical activity. Such situations could be when medical personnel provide them access to concussion management protocols, when concerned parents ask them to quit their sport or when coaches reward playing through pain and injuries.

## 4. Discussion

We identified and described eleven extra-personal concepts influencing concussion reporting across two broad categories: *Contextual Incentives* and *Socio-Cultural Pressures*. This study reports that several extra-personal influences, both contextual and socio-cultural, appear to influence SC reporting by modulating the stakes or perceived costs of disclosure (see Figure 1).

Our results confirm certain concepts that have emerged in previous studies such as the reduced likelihood of concussion disclosure during competition compared to between competitions. They also highlight the critical role of trust in certain sport-specific relationships (i.e., coaches, teammates) and the negative influence of a sport culture that values warrior mentalities and physical toughness, which discourages athletes from disclosing injuries [25,26,28,29,30]. Our work also makes significant contributions by (1) providing a more nuanced understanding of how established concepts influence concussion disclosure, (2) identifying new factors that significantly affect disclosure, and (3) uncovering potential mechanisms through which these factors exert their influence on concussion disclosure.

For example, although all the concepts presented in this study can contribute to the decision-making process, their influence on the context appears to vary, meaning they would not carry equal weight on the decision-making process. According to our data, concepts categorized under Contextual Incentives appear to exert a stronger influence on concussion reporting decisions compared to those under Socio-Cultural Pressures. Notably, contextual incentives, such as athletes’ perceived influence on the outcome of a competition, the subjective importance they ascribe to the moment and specific sport-specific realities, seem to have disproportional influence on athlete’s SC disclosure behavior. Given the exploratory and speculative nature of this observation, future work should investigate this comparison systematically to confirm its validity.

Our findings suggest that the context in which a concussion occurs plays a critical role in influencing disclosure, largely driven by the stakes involved (see Figure 1). Each concept we identified can individually shape the environment around the injury, creating either higher-stakes conditions that deter disclosure or lower-stakes conditions that encourage it. More importantly, our results show that these concepts do not operate in isolation; instead, they interact and collectively influence athletes’ decision-making process by either raising or lowering the perceived stakes of disclosing a concussion.

First, our findings reveal that, when a SC occurs, the subjective importance ascribed to the moment seems to significantly shape athletes’ decisions to disclose their concussion symptoms. While previous studies have noted distinctions between competitive (e.g., games or competitions) and non-competitive (e.g., practices or training) contexts [25,28], our participants went further by making nuanced distinctions within competitive settings. For example, competitions like a league championship or an end-of-season game were viewed as more prestigious and high-stakes compared to regular-season or exhibition games. Participants unanimously reported that high-stakes competitions discouraged concussion disclosure more strongly. This suggests that athletes assess their environments on a graded scale, making relative comparisons between competitive settings, rather than relying on a simple binary evaluation of high-stakes versus low-stakes. As a result, the influence of competition context on concussion disclosure appears to be more complex and multifaceted than previously reported.

Secondly, our data indicate that an athlete’s perceived agency over the outcome of an event may be even more important than the subjective importance of the moment itself. For example, accounts like George’s illustrate that the subjective importance of the moment would matter only when athletes feel that they can exert a meaningful influence on the outcome of the competition. This suggests a specific relationship between two concepts we identified, where the SC non-disclosure probability would be proportional to the product of the athletes’ subjective importance of the moment and the athlete’s perceived influence over its outcome. If further substantiated, this dynamic relationship could be represented using the following equation:

P(non-disclosure) ∝ *Subjective importance of the moment* x *Perceived influence over the outcome of the competition*

This expression illustrates how the disclosure-hindering effect of the importance of the moment can be practically negated when an athlete’s perceived influence over the outcome of the competition approaches zero. To our knowledge, this is the first description of this interaction and of its influence over SC disclosure. As such, future work should explore this relationship to ensure its validity and generalizability. This behavioral calculus seems to align with self-determination theory, particularly through the basic psychological need for competence—defined as the desire to control outcomes and experience mastery [37,38]. When athletes perceive their influence over the outcome as high, they may view a competition as an opportunity to express and validate their competence. Consequently, athletes may choose not to disclose concussion symptoms, fearing they might miss this critical chance. Additionally, the heightened level of challenge and significance attributed to the competition could intensify the athlete’s sense of competence, further raising the stakes of participation [39]. Given evidence suggesting that athletes’ need for competence is greater than the general population, this could also explain why most athletes in our study considered these two contextual variables more influential to the SC disclosure decisional-making process than socio-cultural pressures [40]. However, if an athlete perceives their influence over the competition’s outcome as low, the impact of both contextual variables seems to diminish, allowing other factors, such as social or cultural pressures, to potentially exert more impact on their decision-making process.

Thirdly, our participants reported that, no matter the importance of the moment or their perceived influence over the outcome, disclosure was more likely to occur in non-competitive settings (e.g., during practice or in-between two games) compared to a competitive setting (e.g., an injury occurs during a game). This aligns with previous studies reporting that disclosure is less likely during competitions, which are perceived as higher-stakes situations, compared to non-competitive contexts that are viewed as lower-stakes [25,28]. Several factors have been proposed to explain this behavior, including physiological masking of concussion symptoms by exercise-induced hormones and sympathetic nervous system activity (e.g., adrenaline, endorphins, cortisol) as well as reliance on fast, intuitive thinking, akin to System 1 from Kahneman’s dual-system model [41,42]. While we do not dismiss these hypotheses, our data suggests that, regardless of physiological influences, the context itself also contributes to the disclosure decision-making process, as competitions were universally recognized by participants as higher-stakes environments compared to non-competitive situations.

Additionally, our findings introduce the concept of an athlete’s final season/year as a significant factor within the Contextual Incentives category, revealing that underreporting of concussions is more prevalent during this critical period of their athletic journey. Given athletes in our sample were competing at the Canadian university level, they were eligible to compete for a maximum of five athletic calendar years. Athletes competing in their last season seem to ascribe higher value to their participation in each practice, game, or competition, thereby elevating the baseline level of stakes for all environments in which a sport concussion could occur and modulating their decision-making algorithm towards non-disclosure. This effect on athletes’ behavior resonates with Cialdini’s scarcity principle which states that the value of an object of desire rises as it becomes more scarce [43]. In this context, playing time or having the opportunity to compete is considered as the scarce resource and represents a specific case of Kurtz’s temporal scarcity phenomenon [44]. Consequently, athletes may prioritize participation over health concerns, further complicating the decision to disclose concussion symptoms.

Our identification and description of sport-specific contextual incentives represent another significant original contribution of this study. While previous research has suggested that certain sport-specific norms contribute to higher non-disclosure rates in certain contact sports like ice hockey and American football [16,29], our findings focus on objective structural components inherent to each sport. For instance, certain designs of competitions, such as performing for only a few minutes (e.g., cheerleading) compared to longer durations (e.g., soccer or rugby), or sport-specific limitations, like the difficulty to replace a teammate on short notice because of a challenging barrier to entry (e.g., learning a complex choreography in a short timeframe), can create substantial social and structural pressures against disclosure. This is especially true when placing an athlete on a concussion management protocol incurs significant near-term social and psychological costs on both the athlete and their teammates.

This insight reveals the interplay between sport-specific realities and the broader social consequences they impose within their sporting environment, while also bridging concepts from our Contextual Incentives category with those of the Socio-Cultural Pressures category. Consequently, the combined weight of both contextual and social influences results in consistently high stakes within certain athletic environments. Although this effect was most salient among cheerleaders in our sample, it is reasonable to project this observation to other sports with a similar competitive structure—such as synchronized swimming or diving—where teams perform short yet complex routines in front of judges. Future research should explore these sports and their specific rules and characteristics to determine the broader impact on concussion disclosure, as well as to explore potential regulatory changes that could alleviate this burden.

Our results also suggest that the concepts within the Socio-Cultural Pressures category contribute to the SC disclosure decisional-making process by modulating the stakes surrounding the concussion, albeit to a lesser extent than contextual factors. This may be because much of the perceived socio-cultural pressures of reporting a concussion stems from athletes’ concerns about how their injury might affect others, such as their coaches, teammates or relatives [16,29,30]. In terms of modulating an athlete’s situational stakes, the indirect impact on others is likely secondary to contextual incentives’ more direct influence on the athletes themselves. However, socio-cultural pressures remain important, and our findings not only support these insights but also extend our understanding of how these dynamics interact.

For example, although trust between athletes and team representatives like teammates, coaches and therapists seem to foster SC disclosure, it appears that this beneficial influence can be offset by athletes’ desire to maintain a sense of control over the process. Through the lens of self-determination theory, the automaticity of removing athletes from play, driven by health concerns, may be perceived by athletes as infringing on their basic psychological need for autonomy [37,45]. Thus, trusting relationships can lower the stakes and encourage disclosure, but only if contextual incentives have already significantly lowered the stakes beforehand.

The role of family members and close relatives in the concussion reporting appeared more nuanced and complex compared to coaches and therapists. While some participants admitted that supportive and trusting relationships with family members could foster open communication about health, facilitating disclosure of concussion symptoms, others admitted underreporting or downplaying their symptoms. The latter was often done to shield their family from unnecessary concern or worry. Our data suggest this behavior may be the more prevalent of the two. Resonating with the concept of sport-specific realities, it supports the fact that athletes are highly attuned to the potential consequences of disclosing a sports-related concussion, not only for themselves but also for those close to them.

In the same line, peers, coaches and other authority figures who uphold a sport culture that glorifies a ‘warrior mentality’ and encourages ‘playing through injuries’ can significantly complicate disclosure. From an athlete’s perspective, no doubt the fear of ‘disappointing’ a coach or ‘letting down’ teammates amplify the perceived social cost of reporting a sports-related concussion. This dynamic raises the stakes, as athletes may feel that disclosing their condition could lead to negative judgments or damage their standing within the team.

Lastly, we identified the concept of ‘social media’ as a novel socio-cultural influence on SC disclosure. Although only one participant in our study believed social media significantly impacted SC disclosure decision-making, this participant was among the youngest. This suggests that social media might have a stronger influence on younger athletes and could likely rise in the future. In terms of SC disclosure, social media could act as an amplifier, heightening the stakes by reinforcing existing pressures from teammates, coaches, relatives, and broader narratives of “masculine” or “warrior toughness” pervasive in sports culture. For example, one study found that most athletes tend to use social media differently based on their audience. When sharing for a distant and larger audience, athletes would tend to project an idealized self-image and to share primarily self-enhancing information which could deter SC. However, athletes were more open to share shortcomings or embarrassing information, such as disclosing injuries, when interacting with close friends or relatives (citation: Self-Presentation on social media, Zheng et al. 2020). Given that social media is also known to influence the health behaviors of young adults [46], its role in SC disclosure is worth further exploration.

### Strengths and Limitations

This study contributes significantly to our understanding of SC disclosure. Its main strengths are the identification of novel extra-personal determinants of SC reporting and the description of their specific interactions and contributions to the SC disclosure decision-making process. Detailed accounts such as how an athlete may refrain from reporting a concussion because the championship game is in two days, but then changes their mind and disclose during half-time because their team is losing by an insurmountable margin highlights the subtle, yet noteworthy influence levied by the context in which injuries and disclosure decisions occur. These types of interactions are impossible to capture using closed-ended survey questions and quantitative analysis or when a qualitative investigation is biased towards intra-personal characteristics.

One limitation of our study, common to most qualitative research, involves the potential biases inherent to self-reported data. These can range from inaccurate recalls to purposeful exaggeration of certain beliefs and experiences in an effort to appear socially desirable [19,47]. Thus, the findings reported in this work should be considered exploratory and would benefit from future research using both quantitative and qualitative methods. For example, future qualitative work could try to triangulate the accounts of athletes with those of peers, coaches, medical professionals or relatives who witnessed how they experienced their concussions. In parallel, future quantitative work could investigate the accuracy of some concepts identified in this work by prospectively studying rates of concussion reporting under various settings and contexts.

Another limitation is the small sample size, which was limited to participants representing a narrow age range and specific sport affiliations. Future research should investigate whether concepts identified in our work, and their mechanism of influence, resonate with athletes from more diverse age groups, sports, and cultural backgrounds. For example, compared to our current athlete sample, we hypothesize that concepts within our category of socio-cultural pressures would be quantitatively less influential and qualitatively different for athletes practicing individual sports (tennis, track and field, etc.). Moreover, certain novel concepts identified in our study—such as ‘*social media’*, ‘*athletes’ perceived influence on a competition’s outcome*’, and ‘*sport-specific realities*’—are newly reported determinants of SC disclosure and could benefit from further investigation and elaboration in future research.

Additionally, given the exploratory nature of this work, it could both guide and benefit from future research. For example, insights revealed in this study could guide amendments to existing assessment tools or lead to changes in certain sport regulations to better account for the contextual and socio-cultural influences at play when athletes must report SC symptoms. In turn, these new assessment tools and regulations could be examined and quantitatively compared to previous versions to validate if they indeed improve SC reporting rates.

## 5. Conclusions

This study highlights many of the benefits of investigating concussion disclosure using qualitative methods. Thanks to in-depth interviews, it uncovered several novel modulators of SC disclosure and detailed their mechanistic influence on athlete’s decision-making process. Grounded theory analysis revealed how several extra-personal influences, both contextual and socio-cultural, significantly shape SC reporting by modulating the stakes or perceived costs of disclosure. Notably, contextual incentives such as athletes’ perceived influence on the outcome of a competition, the subjective importance they ascribe to the moment, and specific sport-specific realities seem to have an outsized influence over their disclosure behavior.

If our findings hold true in more diverse populations and contexts, they suggest that adapting concussion prevention efforts to consider these contextual variables could improve SC disclosure. Context-based interventions also offer cost-effectiveness advantages over individualized interventions, as they can benefit all athletes simultaneously. By addressing the broader contextual and socio-cultural factors influencing disclosure, these interventions may create a more supportive environment for athletes to report concussions without fear of repercussions.

## Figures and Tables

**Table 1:** Participant Information.

Participants	University Sport	Age (Years)	History of Concussion (Self-Reported)
Anthony	Soccer	23.17	10
Bianca	Rugby	24.22	3
Hannah	Cheerleading	26.6	3
Inna	Cheerleading	21.6	3
Frank	Soccer	22.12	1
Charles	Soccer	24.66	0
Darya	Cheerleading	25.17	0
Evelyn	Rugby	19.87	0
Georges	Soccer	18.67	0

**Table 2:** Extra-personal concepts and categories influencing concussion disclosure.

Categories	Concepts	Description
**Contextual Incentives**	*Subjective importance of the moment*	Disclosure likelihood seems to be inversely proportional to the athlete’s perceived importance of the moment.
	*Perceived influence on the outcome of the competition*	Athletes seem more inclined to report concussion symptoms when the outcome of the competition is already determined.
	*Timing of decision*	Athletes appear more likely to disclose after or in-between competitions while they would be less likely to disclose concussion symptoms in the middle of a contest.
	*Final season/year*	For most athletes, the sentiment of finality would promote non-disclosure to avoid missing the end of their university career. It could facilitate disclosure in a few athletes looking to avoid significant injuries as they are about to start a job or pursue their career elsewhere.
	*Sport-specific realities*	Sport-specific features such as rules, regulations or structure of the competitions can influence the probability of SC disclosure. For example, sports for which competitions involve short but complex synchronized routines performed in front of judges (ex.: cheerleading) seem to generate stronger incentives against disclosure compared to sports involving two teams competing for the highest number of points/goals (ex.: soccer and rugby).
**Socio-Cultural Pressures**	*Coaches*	The relationship between the coach and the athlete can either hinder or facilitate disclosure depending on its nature and context.
	*Medical Personnel*	Trust in team-specific therapists could favor disclosure, but athletes are wary of their power over the management of their injury.
	*Teammates/Peers*	Influence from teammates and peers seem to promote disclosure, but its real impact on the decisional process may be small.
	*Family/Relatives*	Family and relatives concern over the athletes’ health could hinder concussion disclosure as athletes want to avoid worrying them.
	*Social Media*	Athletes sensitive to social media could be less inclined to report their concussion to avoid disappointing or letting down those looking up to them.
	*General Sport Culture*	Sport cultures rewarding physical toughness and glorifying those who persevere despite pain or injuries would favor non-disclosure.

## Data Availability

The raw data (i.e., full verbatim transcripts of all interviews with study participants) supporting the conclusions of this article will be made available by the authors on request.

## Abbreviations

The following abbreviations are used in this manuscript:

SC	Sports Concussion
GT	Grounded Theory
HoC	History of Concussion
